# CD82 attenuates TGF-β1-mediated epithelial-mesenchymal transition by blocking smad-dependent signaling in ARPE-19 cells

**DOI:** 10.3389/fphar.2022.991056

**Published:** 2022-10-25

**Authors:** Hyesook Lee, Jung-Hwa Han, Yun Jeong Kang, Hyun Hwangbo, Aeseon Yoon, Hyung-Sik Kim, Dongjun Lee, Soo Yong Lee, Byung Hyun Choi, Jae-Joon Kim, Seo Rin Kim, Yung Hyun Choi, Jin Hur

**Affiliations:** ^1^ Department of Convergence Medicine, Pusan National University School of Medicine, Yangsan, South Korea; ^2^ PNU GRAND Convergence Medical Science Education Research Center, Pusan National University School of Medicine, Yangsan, South Korea; ^3^ Department of Biochemistry, Dong-eui University College of Korean Medicine, Busan, South Korea; ^4^ Department of Oral Biochemistry, Dental and Life Science Institute, School of Dentistry, Pusan National University, Yangsan, South Korea; ^5^ Division of Cardiology, Department of Internal Medicine and Research Institute for Convergence of Biomedical Science and Technology, Pusan National University Yangsan Hospital, Yangsan, South Korea; ^6^ Division of Hepato-Biliary-Pancreatic Surgery and Transplantation, Department of Surgery, Pusan National University School of Medicine and Research Institute for Convergence of Biomedical Science and Technology, Pusan National University Yangsan Hospital, Yangsan, South Korea; ^7^ Medical Oncology and Hematology, Department of Internal Medicine, Pusan National University Yangsan Hospital, Yangsan, South Korea; ^8^ Department of Nephrology and Research Institute for Convergence of Biomedical Science and Technology, Pusan National University Yangsan Hospital, Yangsan, South Korea; ^9^ Anti-Aging Research Center and Core-Facility Center for Tissue Regeneration, Dong-eui University, Busan, South Korea

**Keywords:** epithelial-mesenchymal transition (EMT), fibrotic retinal disorders, recombinant human cluster of differentiation 82 (rhCD82), retinal pigment epithelial (RPE), transforming growth factor-beta (TGF-β)

## Abstract

In retinal pigment epithelial (RPE) cells, transforming growth factor-beta (TGF-β) plays a critical role in epithelial-mesenchymal transition (EMT), which contributes to various fibrotic retinal disorders. In the present study, we investigated the effect of recombinant human cluster of differentiation 82 (*rh*CD82), a tumor metastasis suppressor, on TGF-β-induced EMT in the human RPE cell line APRE-19. The results show that TGF-β1 significantly enhanced cell migration, invasion and the expression of EMT-mediate factors in ARPE-19 cells. However, *rh*CD82 markedly inhibited cell mobility and the expression of epithelial marker, zonula occludens-1, as well as increased the expression of mesenchymal markers, such as vimentin and α-smooth muscle actin in TGF-β1-treated APRE-19 cells. In addition, TGF-β1 upregulated the phosphorylation of Smad, extracellular signal regulated kinase (ERK) and glycogen synthase kinase-3β (GSK-3β), but only phosphorylation of Smad was suppressed by *rh*CD82. Noteworthy, *rh*CD82 greatly suppressed the expression of TGF-β receptor I (TGFRI), TGFRII and integrins in TGF-β1-treated APRE-19 cells. In particular, the result of molecular docking analysis and structural modeling show that *rh*CD82 partially interacts with the TGF-β1 binding sites of TGFRI, TGFRII, integrin β1 and integrin αv. Taken together, this finding suggested that *rh*CD82 suppressed TGF-β1-induced EMT of RPE by blocking of Smad-dependent pathway, which is caused by *rh*CD82 interaction with TGFRs and integrins, suggesting new insight into CD82 as a potential therapeutic strategy in fibrotic retinal disorders.

## Introduction

Retinal pigment epithelial (RPE) cells, which are located at the outermost layer of the posterior segment of the eye, maintain the functional integrity of the photoreceptors and choroid and act as a selective barrier (Zhou et al., 2020). Oxidative stress, inflammation and mechanical injury lead to RPE cell damage, which ultimately results in loss of RPE function (Pastor et al., 2002; Choi et al., 2012). RPE dysfunction is involved in the development of various fibrotic retinal disorders, including proliferative vitreoretinopathy (PVR), diabetic retinopathy (DR), and age-related macular degeneration (AMD) (Choi et al., 2012; Zhou et al., 2020). Numerous studies have established that RPE cells undergo epithelial-mesenchymal transition (EMT) during AMD, which is characterized by the acquisition of a phenotype to mesenchymal cells through the loss of cell junction and apical-basal polarity (Takahashi et al., 2010; Radeke et al., 2015). During the EMT process, RPE cells undergo a decrease in junction proteins, such as E-cadherin and zonula occludens-1 (ZO-1), which causes a loss of cell‒cell junctions and an increase in mobility (Gabbiani, 2003). Simultaneously, RPE cells acquire a mesenchymal phenotype through upregulation of N-cadherin, α-smooth muscle actin (α-SMA), and extracellular matrix (ECM) remodeling proteins (Saito, 2013). This EMT process is triggered by multiple extracellular ligands, such as fibroblast growth factor, epidermal growth factor, connective tissue growth factor and insulin-like growth factor-2 and transforming growth factor-beta (TGF-β) (Shu et al., 2020). In particular, among them, TGF-β has been considered a key regulator of EMT in the pathogenesis of RPE (Hinton et al., 2002; Dvashi et al., 2015). TGF-β downstream signaling is initiated through binding to heteromeric TGF-β receptors (Type I and Type II), and subsequently, the TGF-β receptor complex mediates EMT by the canonical Smad or non-Smad pathway (Zhou et al., 2020). In this regard, numerous studies have suggested that blocking TGF-β-mediated EMT through the regulation of Smad-dependent or Smad-independent signaling can be a strategy for developing novel therapeutics for AMD and PVR (Choi et al., 2012; Wei et al., 2018; Chien et al., 2019; Li et al., 2021).

Cluster of differentiation 82 (CD82), also known as KAI1, is a membrane tetraspanin protein family that is expressed in various tissue types (Lee et al., 2021). In many other cancer cell types, CD82 is a well-established tumor metastasis suppressor that represses the functions of motility-related proteins to restrain cell migration and invasion (Liu and Zhang, 2006). Several studies have investigated whether CD82 has a metastasis suppressor effect via crosstalk between TGF-β and Wnt/Smad signaling, which is involved in EMT (Zhang et al., 2019; Lee et al., 2021). Nevertheless, to date, most studies of CD82 have focused on invasive and metastatic cancer, and very few studies have focused on ocular disease (Lee et al., 2021; Ye et al., 2021). In 2021, Ye *et al.* reported that CD82 overexpression induced by intravitreal injection of a rAAV2/9-hsyn-Cd82-2A-mCherry-WPRE-PA vector improved optic nerve axonal transport and axon degeneration in mice (Ye et al., 2021). Additionally, they demonstrated that overexpression of CD82 with rAAV2/9 vector induced optic nerve regeneration in optic nerve crush mice (Ye et al., 2021). More recently, intravitreal injection of recombinant human CD82 (*rh*CD82) protein suppressed retinal neovascularization in oxygen-induced retinopathy (OIR) mice (Lee et al., 2021). Although several studies have suggested that CD82 not only inhibits EMT in various cancers but also attenuates glaucoma and OIR *in vivo*, no studies have established the potential of CD82 in the EMT of RPE cells. Therefore, the aim of the present study is to exploring the role of *rh*CD82 in EMT of REP cells, a hallmark in the pathogenesis of fibrotic retinal disease. In the present study, we elucidate the effect of *rh*CD82 protein on TGF-β-induced EMT and identified the underlying mechanism in the human RPE cell line APRE-19.

## Materials and methods

### Chemicals and reagents

*rh*CD82 protein (catalog No. 12275-H08H) was purchased from Sino Biological Inc. (Beijing, China). A DNA sequence encoding the second extracellular domain of human CD82 (P27701-1) (Gly 111-Leu 228) was fused with a poly-histidine tag at the C-terminus and a signal peptide at the N-terminus. Human recombinant TGF-β1 was obtained from R&D Systems Inc. (Minneapolis, MN, United States).

### Retinal pigment epithelial cell culture

ARPE-19, a human RPE cell line, was obtained from the American Type Culture Collection (Manassas, MD, USA). The cells were grown to 70%–80% confluence and maintained in Dulbecco`s Modified Eagle Medium: Nutrient Mixture F-12 (DMEM/F-12; Invitrogen-Gibco, Carlsbad, CA, United States) supplemented with 10% fetal bovine serum at 37°C in a 5% CO_2_ incubator. ARPE-19 cells from passages 15-20 were used for all experiments. The concentration of 10 ng/ml were selected for TGF- β1, as previous study (Li et al., 2011).

### Cell viability

The viability of cells was measured using a 4-[3-(4-iodophenyl)-2-(4-nitrophenyl)-2H-5-tetrazolio]-1,3-benzene disulfonate (WST-1; Roche Diagnostics, Indianapolis, IN, United States) assay, according to the manufacturer’s instructions. Briefly, the cells were seeded at 1×10^4^ cells/well in 96-well plates for 24 h and pretreated with or without the desired concentration of *rh*CD82 for 1 h. Subsequently, 10 ng/ml TGF-β1 was added for 24 h, and then WST-1 substrate was added to the cells for 2 h. The color development was measured at 450 nm using a microplate spectrophotometer (Genius, Männedorf, Switzerland). The results were expressed as the percentage of the optical density of treated cells relative to that of untreated controls.

### Cell migration assay

Scratch wound closure assays and Transwell invasion assays of confluent ARPE-19 cells were carried out as described previously (Justus et al., 2014; Yu et al., 2017). For the scratch wound closure assay, the wound lines were made by scratching with a 200 μl pipette tip and pretreated with or without *rh*CD82 (100–400 ng/ml) for 1 h, and then 10 ng/ml TGF-β1 was added for 24 h. The width of the wound area was recorded under an inverted microscope (Carl Zeiss, Oberkochen, Germany). For the Transwell invasion assay using a transwell chamber system (Corning, Arizona, United States), 1×10^5^ cells/well were seeded in a Matrigel^TM^ (BD Biosciences, Bedford, MA, United States)-coated insert chamber with 200 µl of serum-free medium. The bottom chamber received 10% FBS-containing DMEM/F-12 medium with or without *rh*CD82 (100–400 ng/ml) and 10 ng/ml TGF-β1. After 48 h, the cells were fixed in 4% poly-methanol for 15 min at room temperature and the invasive cells were stained with 10% Giemsa solution (Sigma–Aldrich Chemical Co., St. Louis, MO, United States) for 20 min at room temperature. Five fields of view were selected at random and observed under a microscope, and stained cells were quantitatively analyzed using the “threshold tool” of ImageJ® (National Institutes of Health, Bethesda, MD, United States).

### Immunoblot analysis

The cells were pretreated with or without *rh*CD82 for 1 h and additionally incubated with 10 ng/ml TGF-β1 for 24 h. As previously described, cells were harvested, and total protein lysates were extracted (Lee et al., 2020). 40 μg of protein were separated by sodium dodecyl sulfate–polyacrylamide gel electrophoresis, transferred onto polyvinylidene difluoride membranes. The membranes were blocked with 5% skim milk in Tris-buffered saline containing 0.1% Tween 20, subsequently probed with primary antibodies overnight at 4°C, and then immunoblotted with the corresponding secondary antibodies for 1 h at room temperature. Information on the antibodies used for immunoblot analysis is summarized in Supplementary Table S1. The membranes were then exposed to enhanced chemiluminescence solution (Thermo Fisher Scientific) and visualized using a LAS-3000 Imaging System (Fujifilm Image Reader, Valhalla, NY, USA).

### Immunofluorescence analysis

Immunofluorescence staining was performed as previously described (Park et al., 2019). After the indicated treatment, the cells were fixed with 4% paraformaldehyde, permeabilized in 0.5%/triton X-100 and then blocked with 2% bovine serum albumin (Sigma-Aldrich Chemical Co.). The cells were probed with anti-ZO-1 (catalog No. 13663, Cell Signaling Technology, Beverly, MA, United States), anti-vimentin (catalog No. sc-6260, Santa Cruz Biotechnology, Santa Cruz, CA, United States), anti-α-SMA (catalog No. 53-9760-82, Thermo Fisher Scientific, Waltham, MA, United States), anti-integrin β1 (catalog No. ab30394, Abcam Inc., Cambridge, United Kingdom), anti-integrin αvβ5 (catalog No. ab177004, Abcam Inc.), anti-TGFβ receptor I (TGFRI; catalog No. sc-518086, Santa Cruz Biotechnology), and anti-TGFβ receptor II (TGFRII; catalog No. orb763093, Biorbyt Ltd., Cambridge, UK) antibodies at 4°C overnight. Subsequently, the cells were incubated with Alexa Fluor 488-labeled goat anti-rabbit immunoglobulin G (IgG; catalog No. A11008, Thermo Fisher Scientific), Alexa Fluor 488-labeled goat anti-mouse IgG (catalog No. A11001, Thermo Fisher Scientific), and Alexa Fluor 568-labeled goat anti-mouse IgG (catalog No. A11004, Thermo Fisher Scientific) antibodies for 1 h in the dark. The cells were counterstained with 4′,6′-diamidino-2-phenylindole (DAPI; Sigma–Aldrich Chemical Co.) at RT for 10 min. After washing cells with PBS, the cells were mounted and visualized by confocal laser scanning microscopy (Carl Zeiss, Oberkochen, Germany).

### Molecular docking and structure modeling

To identify the interacting residues in the protein–protein binding complex between *h*CD82 and integrin αv, integrin β1, and TGF-β receptors, the three-dimensional (3D) structures of TGF-β, integrin αv, integrin β1, and integrin β5 were obtained from the Protein Data Base (PDB; https://www.rcsb.org) (Prlic et al., 2016). Because human TGFRI, TGFRII, and *h*CD82 proteins do not have solved structures in PDB, the 3D structure of these proteins was searched in Alphafold (https://alphafold.ebi.ac.uk) (Varadi et al., 2022). The complexes of *h*CD82/TGFRI, *h*CD82/TGFRII, *h*CD82/integrin β1, and *h*CD82/integrin αv were bound, and the most stable complex was selected from the top 10 complexes obtained from the ZDOCK server (https://zdock.umassmed.edu) (Pierce et al., 2014). The *h*CD82/integrin β5 complex was excluded because the size might be very large, and it was impossible to analyze. The molecular docking complex was analyzed and visualized for the interactions between the proteins using the PyMOL molecular graphics system (Schrodinger, Inc., New York, NY, United States, https://pymol.org).

### Statistical analysis

Data are presented as the mean ± standard deviation. Analysis of variance (ANOVA) and Tukey’s post-hoc analyses were performed for comparisons between groups using GraphPad Prism 5.03 (GraphPad Software Inc., La Jolla, CA, United States). Statistical significance was set at *p* < 0.05.

## Results

### *rh*CD82 has No cytotoxicity in AREP-19 cells

**FIGURE 1:**
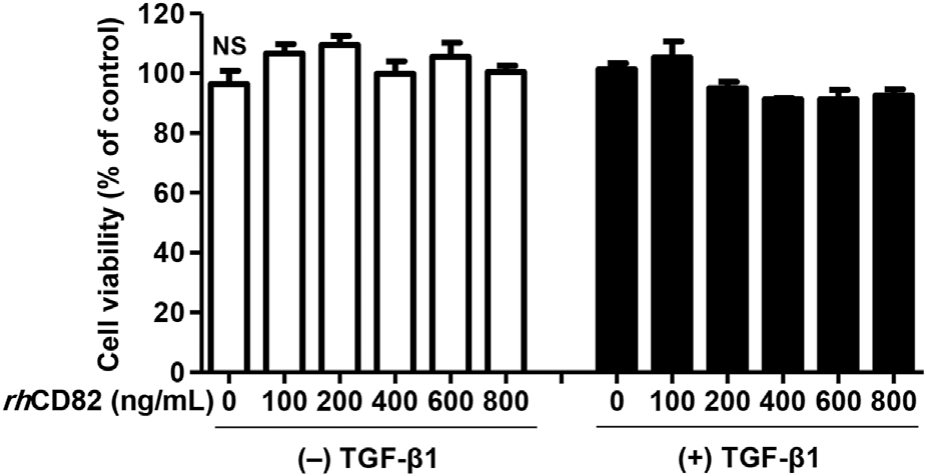
Effect of *rh*CD82 on the viability of ARPE-19 cells. The cells were pretreated with various concentrations (0, 100, 200, 400, 600, and 800 ng/ml) of *rh*CD82 for 1 h before being incubated for 24 h in the presence or absence of 10 ng/ml transforming growth factor (TGF)-β1. Cell viability was measured by WST-1 assay. Data are expressed as the mean ± SD (*n* = 4). NS, no significance.

To select a reasonable concentration of *rh*CD82 to be used for the efficacy evaluation of TGF-β1-mediated cellular changes, ARPE-19 cells were exposed to various concentrations of *rh*CD82 for 24 h. Figure 1 shows that *rh*CD82 had no cytotoxicity at concentrations up to 800 ng/ml in ARPE-19 cells with or without TGF-β1. Treatment with 10 ng/ml TGF-β1 slightly enhanced cell viability, but the level was not significantly different from that in untreated control cells. In addition, pretreatment with *rh*CD82 showed a slight decrease in cell viability in TGF-β1-stimulated ARPE-19 cells, whose levels were similar at concentrations above 400 ng/ml *rh*CD82, whereas these values were also not significantly different from those of control cells.

### *rh*CD82 suppresses the cell migration of transforming growth factor-beta-stimulated AREP-19 cells

**FIGURE 2:**
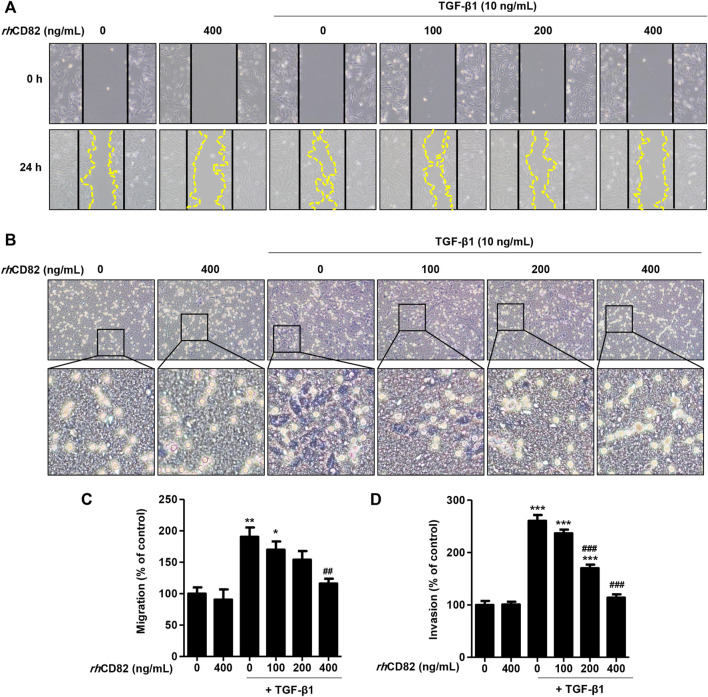
*rh*CD82 suppresses transforming growth factor (TGF)-β1-induced cellular motility in ARPE-19 cells **(A)** Images were acquired at 0 h and 24 h after scratching. Solid lines and dotted lines indicate the edges of the wound and the migrated cells, respectively (magnification, ×100). **(C)** The histogram demonstrates the relative migration ratio compared to the control **(B)** Cells were exposed to 10 ng/ml TGF-β1 in the presence or absence of *rh*CD82 (100–400 ng/ml) for 48 h, and the invaded cells were stained with Giemsa solution. The invaded cells were acquired in five random filters under a microscope at 100× magnification **(D)** Invaded cells were quantitatively analyzed using ImageJ^®^, and the graph shows the relative invasion of cells compared to the control. **(C,D)** Data are expressed as the mean ± SD (*n* = 5). *p < 0.05, **p < 0.01, and ***p < 0.001 compared to control; ^##^ *p* < 0.01 and ^###^ *p* < 0.001 compared to TGF-β1-treated cells.

To investigate the effect of *rh*CD82 on migratory activity, a critical phenotype of EMT, in TGF-β1-stimulated ARPE-19 cells, we performed scratch wound closure and Transwell invasion assays. As a result of the scratch wound closure assay, treatment with TGF-β1 markedly enhanced the migration of cells 24 h after wounding to 190.48% of the control **(** Figures 2A,C). In contrast, pretreatment with *rh*CD82 significantly suppressed the closure in the presence of TGF-β1 in a dose-dependent manner. Similarly, the results of the Transwell invasion assay also showed that TGF-β1 gradually increased the population of invasive cells to 260.47%, of control cells whereas *rh*CD82 markedly decreased TGF-β1-induced cell invasion to 170.07% and 113.98% in a dose-dependent manner (Figures 2B,D).

### *rh*CD82 suppresses transforming growth factor-beta1-induced epithelial-mesenchymal transition in ARPE-19 cells

**FIGURE 3:**
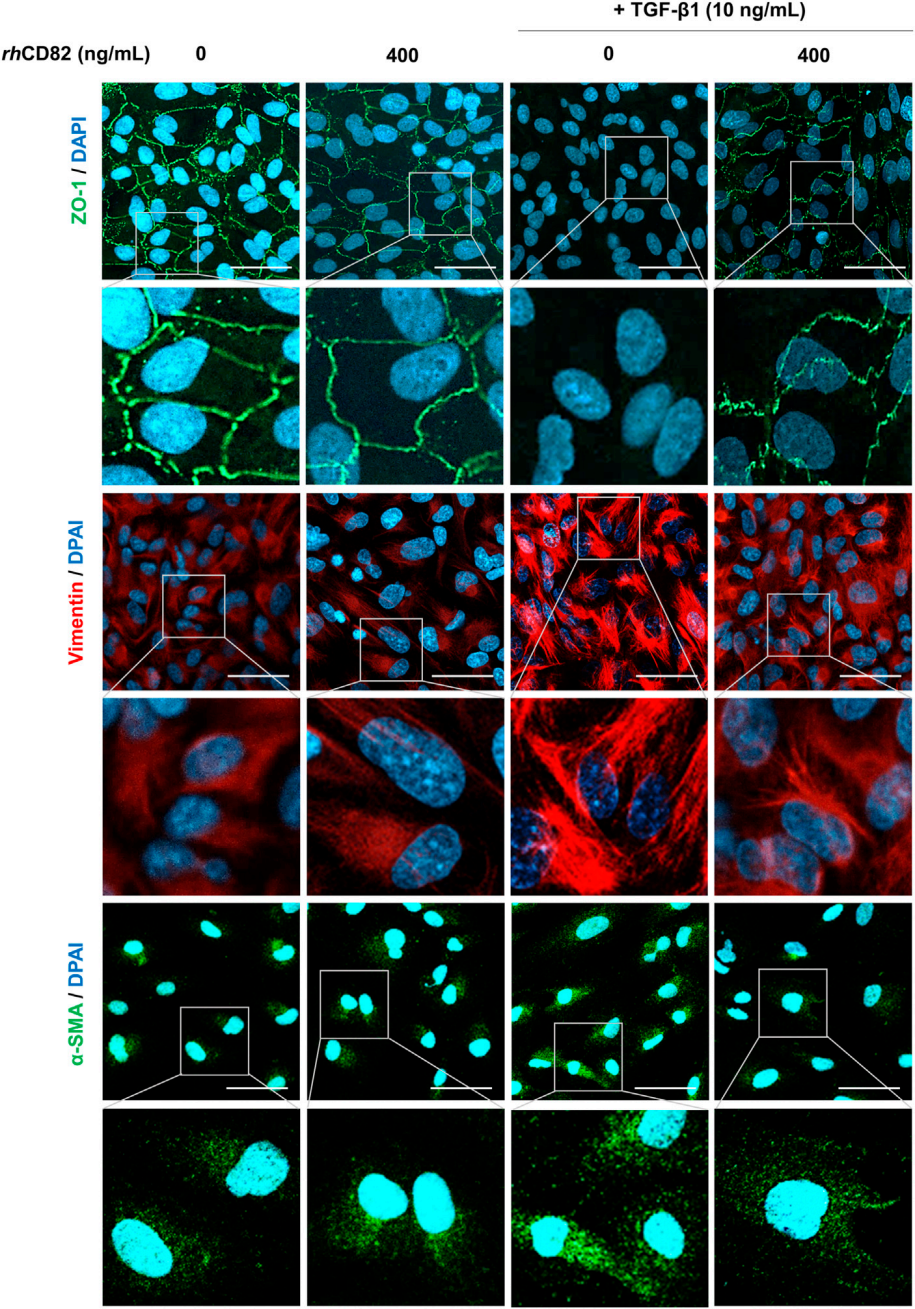
*rh*CD82 suppresses TGF-β1-induced epithelial-mesenchymal transition in ARPE-19 cells. The cells were pretreated with or without 400 ng/ml *rh*CD82 for 1 h and additionally incubated with 10 ng/ml TGF-β1 for 24 h. Subsequently, the cells were immunostained with the epithelial marker zonula occludens-1 (ZO-1; green) and the mesenchymal markers vimentin (red) and α-smooth muscle actin (α-SMA; green). 4′,6′-Diamidino-2-phenylindole (DAPI; blue) was used to counterstain the nuclei. The stained cells were observed under a confocal laser scanning microscope (scale bar, 50 μm).

To assess whether the inhibitory effect of *rh*CD82 on TGF-β1-induced cell migration is associated with the regulation of EMT, we analyzed the expression of EMT markers. Figure 3 shows that the expression of ZO-1, an epithelial marker, was markedly downregulated in TGF-β1-stimulated cells, while it was greatly recovered by pretreatment with *rh*CD82. Furthermore, the expression of mesenchymal markers, including vimentin and α-SMA, was strongly enhanced following TGF-β1 treatment, whereas this overexpression by TGF-β1 was substantially suppressed by *rh*CD82.

### *rh*CD82 attenuates transforming growth factor-beta1-mediated epithelial-mesenchymal transition of ARPE-19 cells through downregulation of the smad-dependent signaling pathway

**FIGURE 4:**
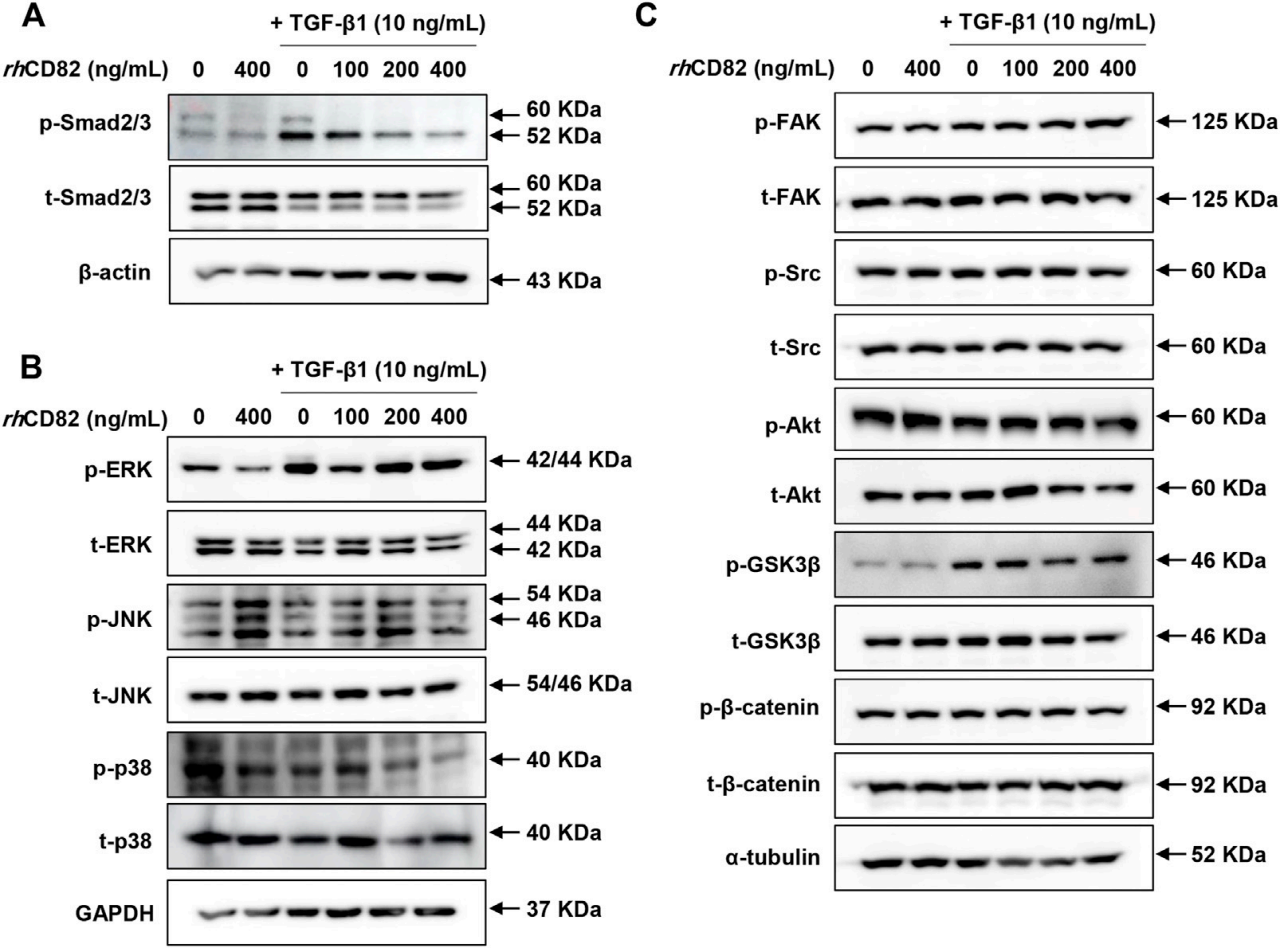
*rh*CD82 attenuates TGF-β1-mediated EMT of ARPE-19 cells through downregulation of the Smad-dependent signaling pathway. The cells were pretreated with or without 400 ng/ml *rh*CD82 for 1 h and additionally incubated with 10 ng/ml TGF-β1 for 24 h. Subsequently, the protein expression of Smad-dependent signaling molecules **(A)**, Smad-independent signaling molecules **(B)**, and integrin-dependent signaling molecules **(C)** was investigated using immunoblot analysis.

To further verify the downstream mechanism by which TGF-β1 regulates EMT and migration, we investigated the expression of TGF-β-involved signaling cascades, including Smad dependent, Smad-independent and integrin-dependent pathways. As shown in Figure 4A, TGF-β1 conspicuously increased the phosphorylation of Smad, whereas the upregulation of p-Smad by TGF-β1 was markedly decreased in *rh*CD8-treated cells. Meanwhile, although the phosphorylation of extracellular signal regulated kinase (ERK) was elevated by TGF-β1, the upregulated expression of p-ERK was not changed by *rh*CD82 (Figure 4B). Furthermore, the expression of total ERK was also not altered in TGF-β1- and *rh*CD82-exposed cells. In addition, the expression and phosphorylation of c-Jun N-terminal kinase (JNK) and p38 were not altered after treatment with TGF-β1 and *rh*CD82. Likewise, the phosphorylation of glycogen synthase kinase-3β (GSK-3β) was also upregulated in TGF-β1-treated cells, while the increase in p-GSK-3β was not reversed in *rh*CD82 (Figure 4C). Moreover, the phosphorylation and expression of focal adhesion kinase (FAK), steroid receptor coactivator (Src), protein kinase B (Akt) and β-catenin did not change following TGF-β1 and *rh*CD82.

### *rh*CD82 inhibits the expression of transforming growth factor-beta1 receptors and integrins in transforming growth factor-beta1-stimulated ARPE-19 cells

**FIGURE 5:**
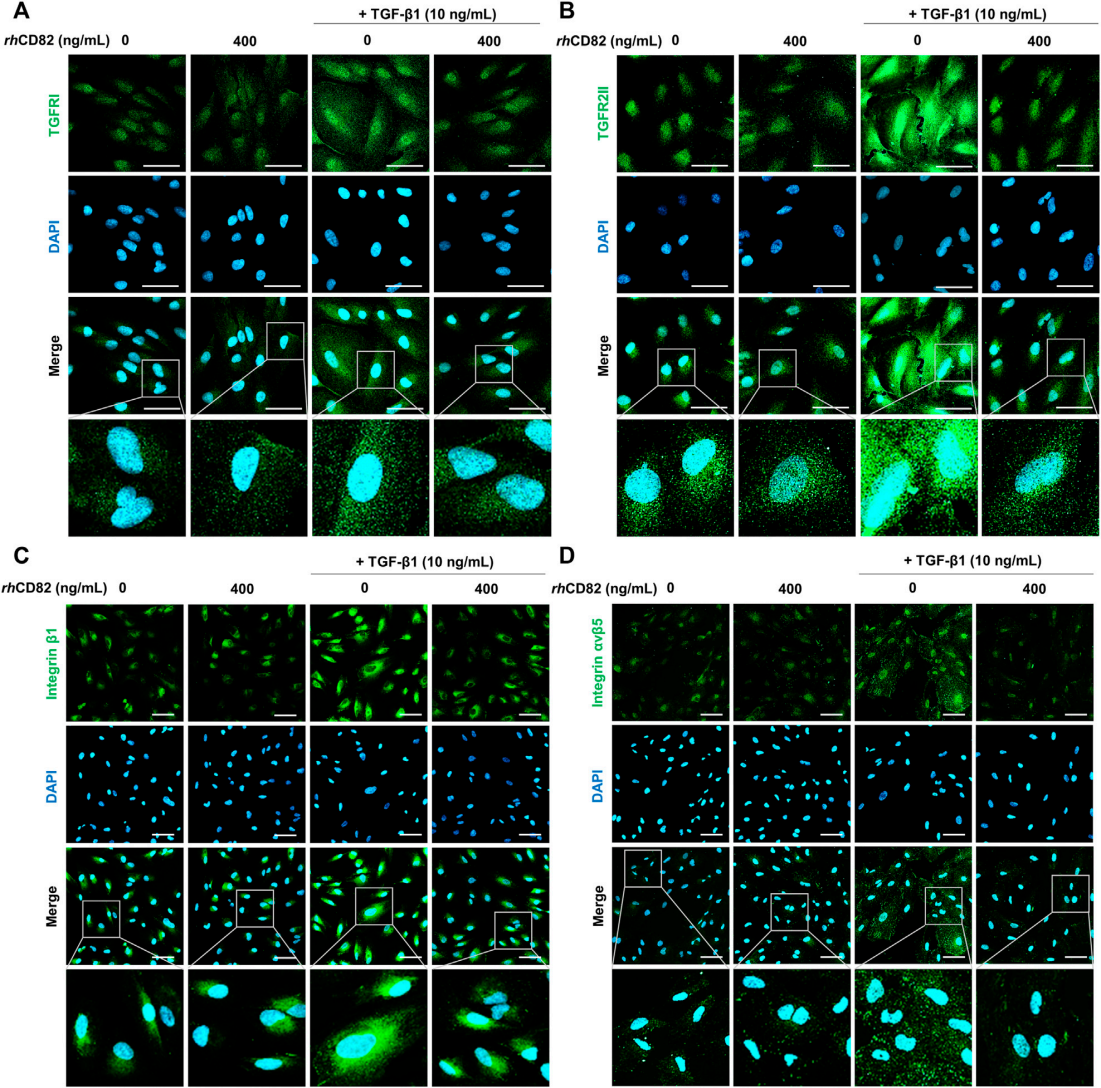
*rh*CD82 inhibits the expression of TGF-β1 receptors and integrins in TGF-β1-stimulated ARPE-19 cells. The cells were pretreated with or without 400 ng/ml *rh*CD82 for 1 h and additionally incubated with 10 ng/ml TGF-β1 for 24 h. Subsequently, the cells were immunostained with TGF-β receptor 1 (TGFR1; **(A)**, TGF-β receptor 2 (TGFR2; **(B)**, integrin β1 **(C)**, and integrin αvβ5 **(D)**. DAPI was used to counterstain the nuclei. The stained cells were observed under a confocal laser scanning microscope (scale bar of A and B, 50 μm; scale bar of C and D, 75 μm).

To evaluate the effect of *rh*CD82 on the expression of integrins and TGF-β receptors following TGF-β1 exposure, we performed immunofluorescence analysis for TGFR1, TGFR2, integrin β1, and integrin αvβ5. Figures 5A,B show that the expression of TGFRs, such as TGFR1 and TGFR2, was strongly increased in TGF-β1-stimulated ARPE-19 cells. In contrast, the overexpression of TGFR1 and TGFR2 by TGF-β1 was notably suppressed by pretreatment with *rh*CD82. This result was consistent with the finding from immunofluorescence analysis for the expression of integrin β1 and integrin αvβ5, as shown in Figures 5C,D.

### *h*CD82 directly interacts with the TGFR1, TGFR1, integrin β1, and integrin αv

**FIGURE 6:**
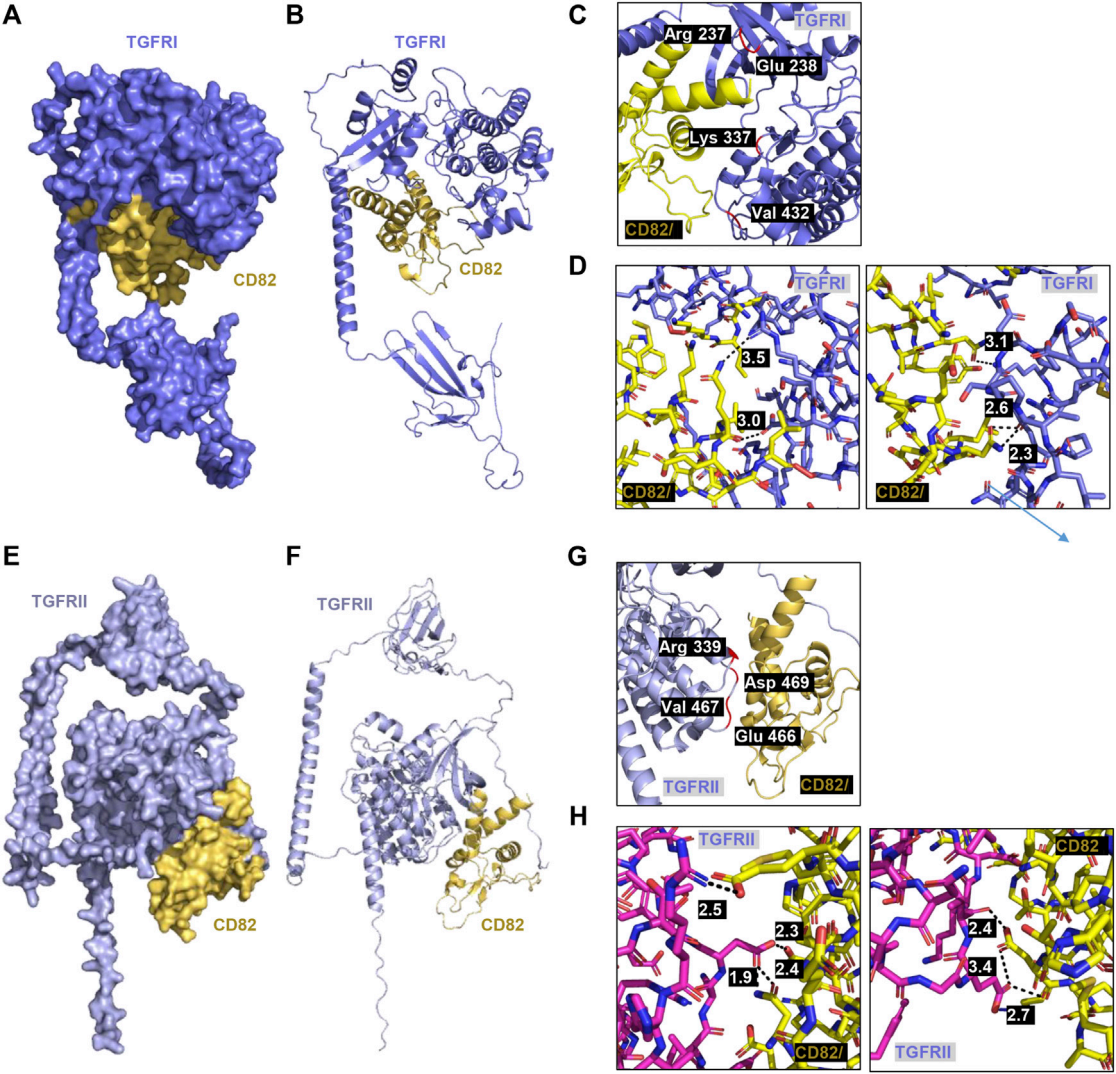
Molecular docking and 3D structure of *h*CD82/TGFRI and the *h*CD82/TGFRII complex. The docking conformations of *h* CD82/TGFRI and *h*CD82/TGFRII are illustrated by the surface **(A,E)** and cartoon **(B,F)**. **(C,G)** The interacting residues of *h*CD82/TGFRI and the *h*CD82/TGFRII complex are shown in r cartoon representation. **(D,H)** The covalent bond of the *h*CD82 (yellow)-TGFRI (dark violet) complex and *h*CD82 (yellow)-TGFRII (pink) is illustrated by a black dotted line, and its distance is presented. Atoms N and O were marked with blue and orange, respectively.

**FIGURE 7:**
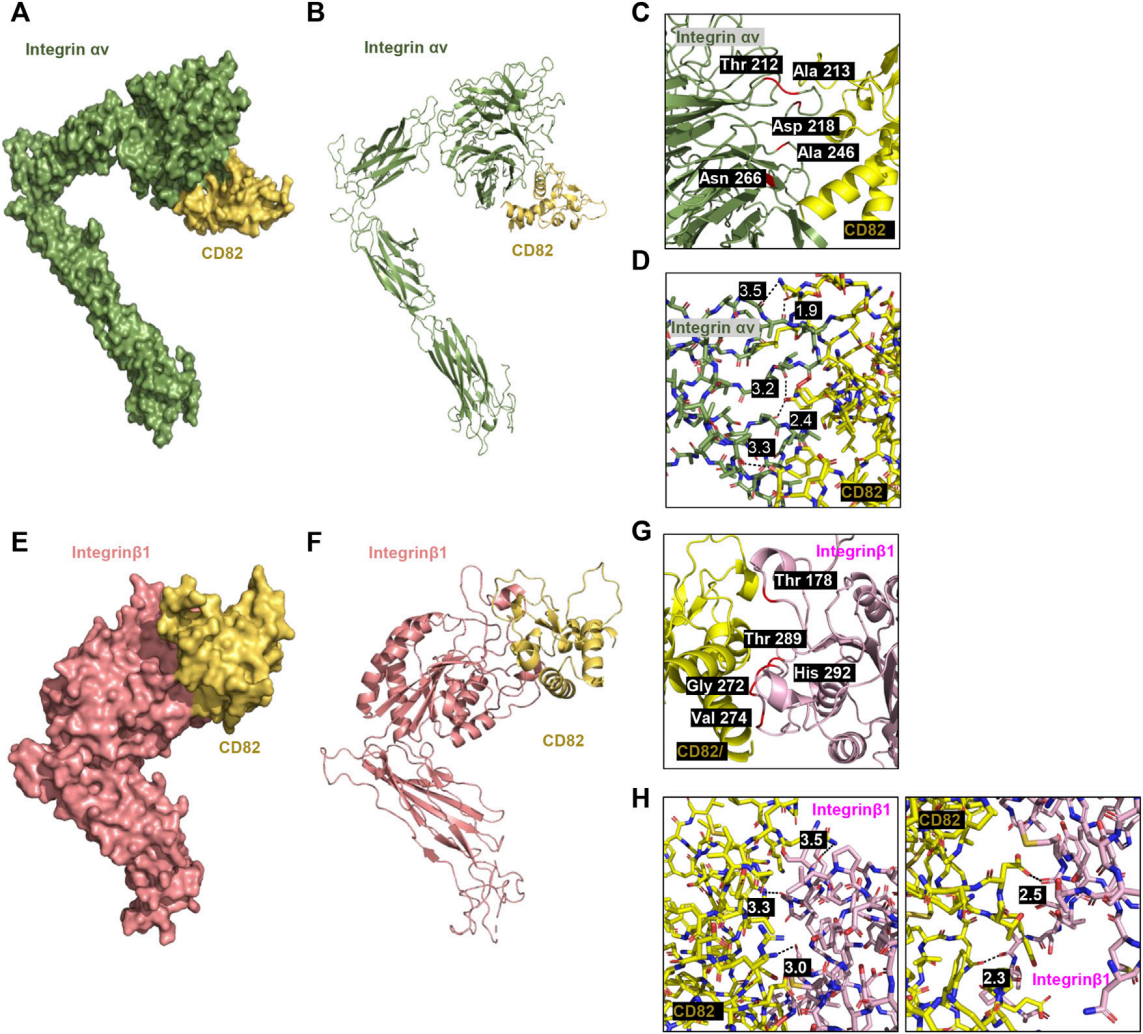
Molecular docking and 3D structure of *h*CD82/integrin αv and the *h*CD82/integrin β1 complex. The docking conformations of *h*CD82/integrin αv and *h*CD82/integrin β1 are illustrated by the surface **(A,E)** and cartoon **(B,F)**. **(C,G)** The interacting residues of *h*CD82/integrin αv and the *h*CD82/integrin β1 complex are shown in cartoon representation. **(D,H)** The covalent bond of the *h*CD82 (yellow)-integrin αv (khaki) complex and*h* CD82 (yellow)-integrin β1 (light pink) is illustrated by a black dotted line, and its distance is presented. Atoms N and O were marked with blue and orange, respectively.

To predict whether *h*CD82 interacts with TGFR and integrins or whether it may prevent the binding of TGF-β1 to TGFR or integrins on the cell surface, we performed molecular docking analysis using the ZDOCK server. As shown in Figures 1, 2, different types of interactions of *h*CD82 with TGFRs and integrins were visualized by PyMOL. Figures 6A,E, Figures 7A,E represent the surface of the protein–protein interaction. In addition, a ribbon cartoon representation of the interacting complex is presented in Figures 6B,F and Figures 7B,F. As a result of the analysis of the interacting residues of the docking complex and its covalent bound distance, *h*CD82 bound to TGFRI in five parts (Figures 6C,D). The first covalent bond between residues Arg 237 of TGFRI and Gln 225 of CD82 was a distance of 3.5 Å (Table 1). The second covalent bond between residues Glu 238 of TGFRI and Leu 224 of *h*CD82 was a distance of 3.0 Å. The third covalent bond between residues Lys 337 of TGFRI and Gln 146 of CD82 was a distance of 3.1 Å. The last two covalent bonds between residues Val 432 of TGFRI and Asn 184 of CD82 were a distance of 2.6 Å and 2.3 Å, respectively. Meanwhile, CD82 interacted with TGFRII in seven regions (Figures 6G,H and Table 1). Among them, the covalent bond between residues Asp 469 of TGFRII and Asn 157 of *h*CD82 had distances of 2.3 Å and 1.9 Å, respectively. Additionally, two covalent bonds between residues Glu 466 of TGFRII and Asp 160 of *h*CD82 were observed at distances of 3.4 Å and 2.7 Å, respectively. Furthermore, the residues Try 412 of *h*CD82 bound with Asp 218 of integrin αv, and the distance of its covalent bond is 3.2 Å (Figures 7C,D and Table 1). Aside from these, *h*CD82 interact with other four integrin αv residues: Thr 212, Ala 213, Ala 246, and Asn 266. Similarly, we found that *h*CD82 interact with five integrin β1 residues: Val 274, Gly 272, His 292, Thr 289, and Thr 178 (Figures 7G,H and Table 1).

**TABLE 1:** The binding information of *h*CD82 with TGFRI, TGFRII, integrin αv and integrin β1.

Complex	Binding site		
CD82/TGFRI	TGFRI (AF ID: P36897)	*h*CD82 (AF ID: P277701)	Distance (Å)
	Arg 237	Gln 225	3.5
	Glu 238	Leu 224	3.0
	Lys 337	Gln 146	3.1
	Val 432	Asn 184	2.6
	Val 432	Asn 184	2.3
CD82/TGFRII	TGFRII (AF ID: P37173)	*h*CD82 (AF ID: P277701)	Distance (Å)
	Arg 339	Glu 218	2.5
	Asp 469	Thr 122	2.3
	Asp 469	Asn 157	2.3
	Asp469	Asn 157	1.9
	Val 467	Asp 160	2.4
	Glu 466	Asp 160	3.4
	Glu 466	Asp160	2.7
CD82/Integrin αv	Integrin αv (PDB ID: 6AVR)	*h*CD82 (AF ID: P277701)	Distance (Å)
	Thr 212	Asn 184	3.5
	Ala 213	Asn 184	1.9
	Asp 218	Tyr 142	3.2
	Ala 246	Tyr 142	2.4
	Asn 266	Asn 227	3.3
CD82/Integrin β1	Integrin β1 (PDB ID: 3VI4)	*h*CD82 (AF ID: P277701)	Distance (Å)
	Val 274	Gln 115	3.5
	Gly 272	Gly 118	3.3
	His 292	Asn 157	3.0
	Thr 289	Asp160	2.5
	Thr 178	Gln 213	2.3

The complexes of *h*CD82/TGFRI, *h*CD82/TGFRII, *h*CD82/integrin β1, and *h*CD82/integrin αv were bound, and the most stable complex was obtained from the ZDOCK, server. The interacting residues and distances of covalent binding for *h*CD82/TGFR, and hCD82/integrin complexes are presented.

## Discussion

Tetraspanins are expressed on the intracellular membrane and cell surface and control various biological events, including antigen presentation, cell signal transduction, adhesion, migration and motility (Yan et al., 2021). CD82, one of the established human tetraspanins, is well known as a tumor metastasis suppressor gene in various types of cancers (Feng et al., 2015; Yan et al., 2021). CD82 consists of two extracellular loops with its N- and C-termini, four transmembrane domains and a small inner cytoplasmic loop (Mazurov et al., 2007; Hochheimer et al., 2019). Importantly, the large extracellular loop (LEL; Gly 111-Leu 228) of *h*CD82 interacts with specific membrane partners, such as integrins, and is essential for the biological activities of tetraspanins (Wright et al., 2004; Ho et al., 2006; Mazurov et al., 2007). Interestingly, a recent study demonstrated that recombinant protein for LEL of *h*CD82 directly bound to vascular endothelial growth factor (VEGF) and platelet-derived growth factor (PDGF), resulting in antiangiogenic and antimetastatic effects (Lee et al., 2021). Additionally, noteworthy, most retinal disorders, including AMD, DR, and retinopathy of prematurity, are involved in inflammation, angiogenesis, and EMT-driven fibrosis, which is accompanied by increased motility (Dreyfuss et al., 2015).

Accumulated evidence has shown that CD82 is a potent tumor suppressor (Yan et al., 2021). Numerous studies have reported that CD82 is downregulated in malignant tumors of various organs, including the lung, breast, ovary, and liver (Feng et al., 2015; Yan et al., 2021). Currently, Lee *et al* demonstrated that administration of the mimic full-length LEL of CD82 and the selective 10-mer peptide of CD82 LEL markedly suppressed tumor angiogenesis and growth in melanoma, prostate cancer and pancreatic carcinoma (Lee et al., 2021). Furthermore, another recent study suggested that a peptide mimicking the extracellular loop of CD82 inhibited metastasis, invasion, and adherence in *in vitro* and *in vivo* pulmonary carcinoma models (Valcourt et al., 2005). Based on these previous studies showing that the anticancer effect of LEL of CD82 is involved in a potential ability to suppress motility and angiogenesis, we hypothesized that its capacity may contribute to protecting against fibrotic retinal disease. Here, we evaluated the effect of *rh*CD82 protein, a protein mimicking the LEL domain of *h*CD82 on TGF-β-induced EMT and identified the underlying mechanism in the human RPE cell line APRE-19. Our findings showed that *rh*CD82 had significant inhibitory effects on cell migration and invasion in TGF-β1-exposed ARPE-19 cells, and the most significant blockage of motility was noted at a *rh*CD82 concentration of 400 ng/ml, which was similar to the efficacy of the control. Moreover, we found that TGF-β1-stimulated ARPE-19 cells underwent EMT through the acquisition of mesenchymal markers and the loss of epithelial markers, while this TGF-β1-mediated EMT progression was noticeably reversed by pretreatment with *rh*CD82. In the Smad-dependent pathway, TGF-β ligand and the TGF-β receptor complex induce phosphorylation of Smad2 and Smad3 and ultimately promote the transcription of several EMT-regulated genes through nuclear translocation of the Smad complex (Valcourt et al., 2005). Smad-mediated TGF-β signaling can activate integrin-linked kinase (ILK), which interacts with the cytoplasmic domains of integrins and cytoskeletal proteins and leads to the phosphorylation of GSK-3β/β-catenin (Willis and Borok, 2007; Mamuya and Duncan, 2012). Meanwhile, TGF-β also stimulates the noncarnonical Smad-independent pathway, including mitogen-activated protein kinase (MAPK), phosphatidylinositol-3-kinase/Akt (PI3K/Akt), and Wnt-β-catenin (Han et al., 2015; Luo, 2017). Our results showed that although TGF-β1 upregulated the phosphorylation of Smad, Smad-independent ERK and integrin-dependent GSK-3β, among them, the phosphorylation of Smad was only downregulated by *rh*CD82. This finding indicated that the Smad signaling pathway was involved in the suppression of TGF-β1-mediated changes in the EMT phenotype by *rh*CD82 in ARPE-19 cells. Several previous studies also demonstrated that CD82 represses TGF-β1-mediated EMT through regulation of different signaling pathways in various carcinoma types (Zhu et al., 2017; Zeng et al., 2018; Lee et al., 2019). Lee *et al.* reported that CD82 downregulated the Wnt signaling pathway, resulting in TGF-β1-mediated EMT in human prostate cancer cells (Lee et al., 2019). In 2017, Zhu *et al.* demonstrated that CD82 inhibits cell migration and invasion *via* TGF-β 1/Smad signaling in renal cell carcinoma (Zhu et al., 2017). Zeng *et al.* also suggested that CD82 inhibits the invasion and metastasis of esophageal squamous carcinoma cells by blocking the TGF-β 1/Smad2/3 signaling pathway (Zeng et al., 2018).

As mentioned earlier, TGF-β has been considered a key regulator of EMT in the pathogenesis of RPE, which actives binding to the heteromeric TGFRI/TGFRII complex (Zhou et al., 2020). Moreover, several αV integrins are over-expressed during EMT process, but it is expressed at low levels in healthy epithelium (Mamuya and Duncan, 2012). There has been reported that integrins αvβ3, αvβ5, and αvβ6 were all shown to activate TGF-β1 by transmitting cell forces to the latent TGF-β1 complex (Sarrazy et al., 2014). Furthermore, TGF-β signal transduction can be stimulated by binding with integrin αvβ6, which is highly expressed on damaged epithelial cells and carcinoma cells *in vitro* and *in vivo* (O'Connor and Gomez, 2014; Munger et al., 1999). Based on these findings, to evaluate the potential of CD82 to bind TGFRs and integrins, which are key transmembrane regulators of TGF-β1 in the RPE, we performed molecular docking analysis using the ZDOCK server. Moreover, we estimated whether it prevents the binding of TGF-β1 to TGFR or integrins on the cell surface. In the current study, we confirmed that the TGF-β1-mediated EMT process is accompanied by upregulation of TGFRI, TGFRII, integrin β1 and integrin αvβ5 in ARPE-19 cells, whereas this event is suppressed in the presence of *rh*CD82. Additionally, we found that the LEL domain of *h*CD82 bound with residues 237-432 of TGFRI and residues 339-466 of TGFRII containing protein kinase domain (Chaikuad and Bullock, 2016). Furthermore, our finding shown that Thy 142 residue of *h*CD82 directly interact with Asp 218 residue of integrin αv which is the residue bind with Arg 215 of TGF-β1 (Shi et al., 2011; Dong et al., 2017). These results suggest that the LEL domain of *h*CD82 may directly or indirectly inhibit the binding of TGF-β1 or the TGFRI/II and integrins, resulting TGF-β downstream signal pathway.

**FIGURE 8:**
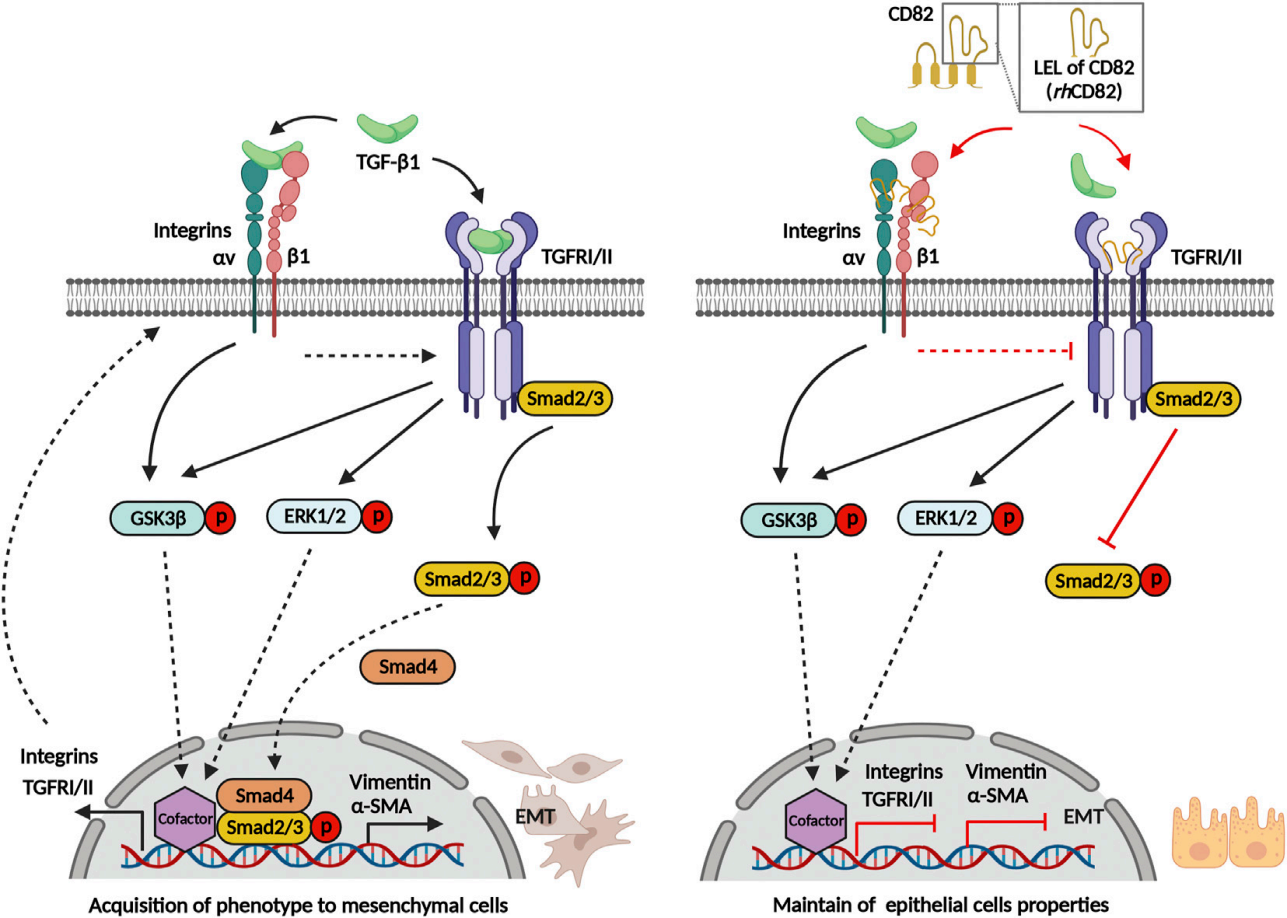
Graphical abstract of *rh*CD82 suppressing TGF-β1-mediated epithelial-mesenchymal transition by blocking the Smad-dependent signal in ARPE-19 cells, which is due to interaction with integrin and TGF-β receptor.

Overall, our present findings showed that *rh*CD82, a partial protein of human CD82 LEL, suppressed TGF-β1-induced EMT of RPE, including increasing migration and invasion, acquisition of mesenchymal phenotype, and overexpression of TGFRs and integrins (Figure 8). This inhibitory effect of *rh*CD82 on TGF-β1-mediated EMT is associated with the LEL domain of CD82 interacting with TGFR and integrins, and it may prevent the binding of TGF-β1 to TGFR or integrins on the cell surface, which leads to blockade of TGF-β signal transduction via the Smad-dependent pathway. However, the limitations of the present study are the lack of the result for direct interaction with *rh*CD82 and TGFR and integrins using immunoprecipitation and blitz techniques, although we predicted the interaction using molecular docking and structure modeling. In addition, we considered that the further studies are warranted to identify the effect of *rh*CD82 on retinal disorders *in vivo* system. Although further studies still remain to be addressed, our findings suggest the important evidence that the effect of rhCD82 against to the TGF-β1-mediated EMT, and new insight into *rh*CD82 as a potential therapeutic strategy in fibrotic retinal disorders.

## Data Availability

The original contributions presented in the study are included in the article/Supplementary Material, further inquiries can be directed to the corresponding authors.
