# Supplementary material for: CD82 attenuates TGF-β1-mediated epithelial-mesenchymal transition by blocking smad-dependent signaling in ARPE-19 cells
Source: Front Pharmacol. 2022 Oct 25;13:991056. doi: 10.3389/fphar.2022.991056 (PMC9640495; doi:10.3389/fphar.2022.991056)
Supplement: Supplementary file 1 [file Table1.docx]

**Supplementary Table S1.** Primary and secondary antibodies used for immunoblotting.

| **Antibodies** | **Supplier** | **Catalog No.** | **Dilution** |
| --- | --- | --- | --- |
| Akt | Cell signaling Technology | #4691 | 1:1,000 |
| β-actin | Santa Cruz Biotechnology, Inc. | sc-47778 | 1:2,000 |
| β-catenin | Cell signaling Technology | #8480 | 1:1,000 |
| ERK | Cell signaling Technology | #4377 | 1:1,000 |
| FAK | Cell signaling Technology | #3285 | 1:1,000 |
| GAPDH | Santa Cruz Biotechnology, Inc. | sc-365062 | 1:2,000 |
| GSK3β | Cell signaling Technology | #12456 | 1:500 |
| JNK | Santa Cruz Biotechnology, Inc. | sc-7345 | 1:1,000 |
| p38 MAPK | Cell signaling Technology | #9212 | 1:1,000 |
| Smad2/3 | Cell signaling Technology | #8685 | 1:500 |
| Src | Cell signaling Technology | #2109 | 1:500 |
| phospho-Akt | Cell signaling Technology | #4060 | 1:1,000 |
| phospho-β-catenin | Cell signaling Technology | #8814 | 1:1,000 |
| phospho-ERK | Cell signaling Technology | #4370 | 1:1,000 |
| phospho-FAK | Cell signaling Technology | #3281 | 1:1,000 |
| phospho-GSK3β | Cell signaling Technology | #5558 | 1:500 |
| phospho-JNK | Santa Cruz Biotechnology, Inc. | sc-6254 | 1:1,000 |
| phospho-p38 MAPK | Cell signaling Technology | #9215 | 1:1,000 |
| phospho-Smad2/3 | Cell signaling Technology | #8828 | 1:500 |
| phospho-Src | Cell signaling Technology | #2105 | 1:500 |
| goat anti-mouse IgG-HRP | Santa Cruz Biotechnology, Inc. | sc‑2005 | 1:2,500 |
| goat anti-rabbit IgG-HRP | Santa Cruz Biotechnology, Inc. | sc‑2004 | 1:2,500 |

Cell Signaling Technology (Beverly, MA, USA); Santa Cruz Biotechnology, Inc. (Santa Cruz, CA, USA)
